# Crystal structure of Ti_8_Bi_9_O_0.25_ containing inter­stitial oxygen atoms

**DOI:** 10.1107/S205698901801188X

**Published:** 2018-08-24

**Authors:** Hisanori Yamane, Keita Hiraka

**Affiliations:** a Institute of Multidisciplinary Research for Advanced Materials, Tohoku University, 2-1-1 Katahira, Aoba-ku, Sendai, 980-8577, Japan

**Keywords:** titanium bis­muth oxygen, Bi flux growth, crystal structure, inter­stitial site

## Abstract

Single crystals of Ti_8_Bi_9_O_0.25_, titanium bis­muth oxide (8/9/0.25), were obtained from a sample prepared by heating a mixture of Ti, TiO_2_ and Bi powders in an Ar atmosphere. Single-crystal X-ray analysis revealed that the introduction of O atoms into the structure of Ti_8_Bi_9_ retains the space-group type *P*4/*nmm*. The oxygen site is located within a Ti_4_ tetra­hedron (point group symmetry  *m*2) that is vacant in the Ti_8_Bi_9_ crystal structure. The occupancy of this site is 0.25 (4), and the O—Ti distance is 1.8824 (11) Å.

## Abstract 2

Single crystals of Ti_8_Bi_9_O_0.25_ were grown by using a Bi flux and were investigated in order to clarify the structural changes by O atom occupation at the inter­stital site in the Ti_4_ tetra­hedron.

## Chemical context   

The crystal structure of Ti_8_Bi_9_, having the tetra­gonal space group *P*4/*nmm, a* = 10.277 (1) Å, *c* = 7.375 (1) Å, *Z* = 2, was determined by Richter and Jeitschko (1997 ▸). This compound was identified in Ti–Bi binary phase diagrams (Okamoto, 2010 ▸, 2015 ▸), and was also confirmed by powder X-ray diffraction (PXRD) in a study of the Ti–Bi phase diagram (Maruyama *et al.*, 2013 ▸). Recently, the use of a Bi flux has enabled single-crystal growth of a new polymorph of TiO (*∊*-phase; Amano *et al.*, 2016 ▸) and some new suboxides: Ti_8_(Sn_*x*_Bi_1–*x*_)O_7_, Ti_11.17_(Sn_0.85_Bi_0.15_)_3_O_10_ and Ti_12–*δ*_Ga_*x*_Bi_3–*x*_O_10_ (Amano & Yamane, 2017 ▸; Yamane & Amano, 2017 ▸). While exploring new suboxides containing Ti using a Bi flux, we also found the title compound, Ti_8_Bi_9_O_0.25_ where inter­stitial O sites are partly occupied.

In the present communication, details of single-crystal growth of Ti_8_Bi_9_O_0.25_ and its comparison with the crystal structure of Ti_8_Bi_9_ (Richter & Jeitschko, 1997 ▸) are reported.

## Structural commentary   

Reflections from a single crystal of Ti_8_Bi_9_O_0.25_ could be indexed with a primitive tetra­gonal cell similar to that of the oxygen-free compound Ti_8_Bi_9_ (Richter & Jeitschko, 1997 ▸). The differences in the lengths of the *a* and *c* axes and in the cell volume from those of Ti_8_Bi_9_ were +1.0%, −0.09% and +0.74%, respectively. The reflection conditions observed for the new compound were the same as for Ti_8_Bi_9_, revealing space group *P*4/*nmm.*

**Figure 1:**
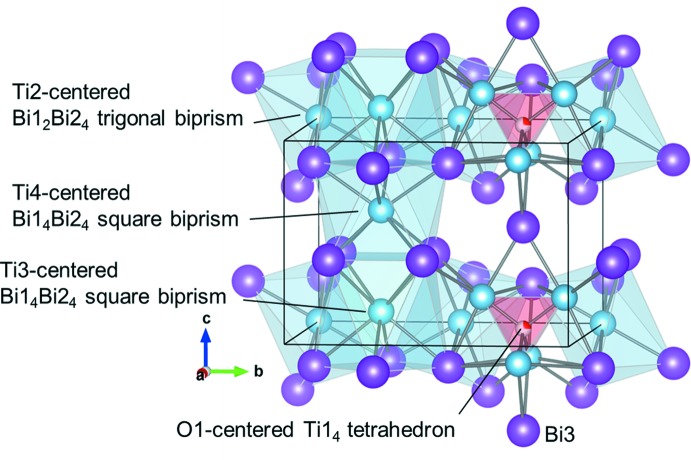
Crystal structure of Ti_8_Bi_9_O_0.25_ drawn with Ti- and O-centered Bi polyhedra. The red part of the O1 sphere in the Ti1_4_ tetra­hedron shows the occupancy.

The crystal structure and atomic arrangement for Ti_8_Bi_9_O_0.25_ are depicted in Figs. 1 ▸ and 2 ▸, respectively. In the crystal structure of Ti_8_Bi_9_ (Richter & Jeitschko, 1997 ▸), the Ti2 site is in a trigonal anti­prism (point group symmetry. .2/*m*) made up from Bi atoms with Bi—Ti distances of 2.848 (1) and 2.931 (1) Å (Table 1 ▸). The Ti3 and Ti4 sites are situated in square anti­prisms in which the Bi—Ti distances range from 2.937 (5) to 3.144 (6) Å. The Ti3- and Ti4-centered Bi1_4_Bi2_4_ square anti­prisms both exhibit point group symmetry 4*mm* and are arranged along the *c* axis by sharing the square planes. The Bi1Bi2_2_ triangle plane is shared by the Ti2-centered Bi1_2_Bi2_4_ trigonal anti­prism and the Ti3-centered Bi1_4_Bi2_4_ square anti­prism. In the crystal structure of Ti_8_Bi_9_, only the Ti1 site forms a Ti polyhedron. The Ti1—Ti1 distances of the Ti1_4_ tetra­hedron are 2.934 (6) and 3.074 (3) Å. In addition to the three Ti1 sites, each Ti1 site is surrounded by six Bi atoms at distances of 2.945 (4)–3.074 (5) Å, and by two Ti2 sites at a distance of 3.017 (2) Å. The O atom of Ti_8_Bi_9_O_0.25_ is located in the Ti1_4_ tetra­hedron at a site with symmetry 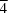 *m*2 and with a site occupancy of 0.25 (4). The partial occupation by the O atoms changes the Ti1—Ti1 distances in the tetra­hedron to 2.992 (2) and 3.1142 (19) Å, representing increases of 1.9% and 1.3%, respectively. The Ti1—Bi2 distance is also increased by 1.4%, although the changes in the Ti3—Bi and Ti4—Bi distances are both less than 0.4%.

The O1—Ti1 distance of 1.8824 (11) Å is inter­mediate between the sums of ionic radii for Ti^3+^ and O^2−^ (1.91 Å) and Ti^2+^—O^2−^ (1.845 Å), based on ionic radii of 0.67 and 0.605 Å for Ti^3+^ and Ti^2+^, respectively, in sixfold coordination, and an O^2−^ radius of 1.24 Å in fourfold coordination (Shannon, 1976 ▸). The bond-valence sums (BVSs) calculated for the O1 site in the Ti1_4_ tetra­hedron using bond-valence parameters (*R*_0_) for Ti^4+^ (1.815 Å), Ti^3+^ (1.815 Å) and Ti^2+^ (1.734 Å) and *B* = 0.37 (Brese & O’Keeffe, 1991 ▸; Amano & Yamane, 2017 ▸) are 3.33, 3.12 and 2.68 valence units (v.u.), respectively. All of these values are considerably greater than the expected valence value of 2 for an O atom, which may suggest that the O1 site is not fully occupied, or that bond-valence parameters for titanium in lower oxidation states (and/or tetra­hedral coordination) need revision. Complete occupation of O atoms in tetra­hedral sites surrounded by Ti atoms has been reported for the crystal structures of Ti_12-*δ*_Ga_*x*_Bi_3–*x*_O_10_. In these structures, the Ti—O distances range from 1.957 (3) to 2.291 (3) Å, all of which exceed the value of 1.8824 (11) Å for O1—Ti1 in Ti_8_Bi_9_O_0.25_. The BVSs calculated for the O sites in Ti_12–*δ*_Ga_*x*_Bi_3–*x*_O_10_ using the parameters for Ti^3+^ and Ti^2+^ were found to be in the ranges 2.18–2.21 and 1.87–1.89 v.u., respectively (Amano & Yamane, 2017 ▸).

## Synthesis and crystallization   

A sample containing the title compound was prepared by combining 0.85 mmol Ti powder (99.99%, Mitsuwa Chemical Co., Ltd), 0.125 mol TiO_2_ powder (rutile, 99.99%, Rare Metallic Co., Ltd) and 1.5 mmol Bi powder (99.999%, Mitsuwa Chemical Co., Ltd) in an agate mortar and subsequent pressing into a pellet (Ø 6 mm) under atmospheric conditions. The pellet was placed in a Ta boat that was then transferred into a stainless-steel tube and sealed with a cap in an Ar-filled glove box (MBRAUN; O_2_ and H_2_O < 1 ppm). The sealed stainless-steel tube was heated to 1073 K at a rate of approximately 400 K h^−1^, maintained at this temperature for 10 h, and subsequently cooled to 723 K at a rate of 10 K h^−1^. Below 723 K, the sample was cooled to room temperature by shutting off the electric power to the heater of the furnace. The resulting sample was crushed and single-crystal fragments of Ti_8_Bi_9_O_0.25_ were extracted. A single crystal for XRD analysis was sealed in a glass capillary. The crushed sample was also analyzed by electron probe microanalysis (EPMA, JEOL, JXA-8200). Only Bi, Ti and O were found in the bulk. The O concentration was greater than the expected values, indicating that some oxidation had occurred while transferring the specimens to the EPMA instrument. In addition to fragments with a Ti:Bi atomic ratio of approximately 8:9, some Bi-rich (>85%) portions and fragments with a Ti:Bi ratio of approximately 3:2 were also identified.

## Refinement   

Crystal data, data collection and structure refinement details are summarized in Table 2 ▸. The diffraction data of Ti_8_Bi_9_O_0.25_ were initially analyzed using the Ti_8_Bi_9_ model (Richter & Jeitschko, 1997 ▸), and a residual electron density of 8.4 e Å^−3^ was observed at (3/4, 1/4, 0), which corresponds to the 2*a* site in the Ti1_4_ tetra­hedron. The O-atom occupancy of this site was refined to be 0.25 (4), resulting in a decrease in *R*[*F* ^2^ > 2*σ* (*F* ^2^)] from 0.045 to 0.020. For this site an isotropic atomic displacement parameter was considered.

## Supplementary Material

Crystal structure: contains datablock(s) I, Ti8Bi9O0.25. DOI: 10.1107/S205698901801188X/wm5456sup1.cif

Structure factors: contains datablock(s) I. DOI: 10.1107/S205698901801188X/wm5456Isup4.hkl

CCDC reference: 1863407

Additional supporting information:  crystallographic information; 3D view; checkCIF report

## Figures and Tables

**Figure 2:**
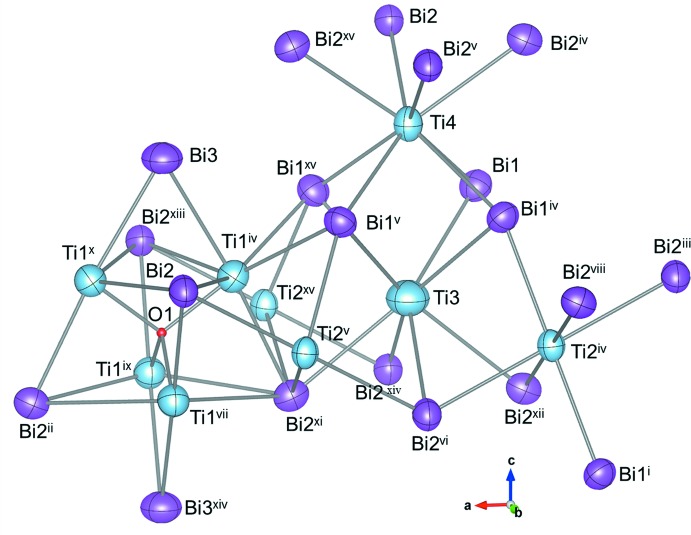
Atomic arrangement around Ti and Bi atoms in the structure of Ti_8_Bi_9_O_0.25_. Displacement ellipsoids are drawn at the 99% probability level. [Symmetry codes: (i) −*y*, −*x* + 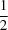, −*z*; (ii) *y* + 1, −*x* + 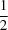, *z* − 1; (iii) −*x* + 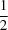, −*y* + 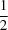, *z* − 1; (iv) *y*, −*x* + 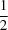, *z*; (v) −*x* + 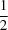, −*y* + 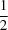, *z*; (vi) −*x* + 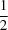, −*y* + 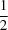, *z* − 1; (vii) −*x* + 1, −*y* + 1, -*z;* (viii) −*y*, *x* + 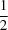, −*z* + 1; (ix) *x* + 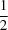, *y* − 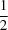, −*z*; (*x*) −*y* + 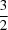, *x*, *z*; (xi) −*y* + 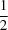, *x*, *z* − 1; (xii) *y*, −*x* + 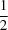, *z* − 1; (xiii) *x* + 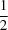, *y* − 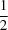, −*z* + 1; (xiv) *x*, *y*, *z* − 1; (xv) −*y* + 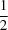, *x*, *z*.]

**Table 1:** Selected inter­atomic distances (Å) for Ti_8_Bi_9_ (Richter & Jeitschko, 1997 ▸) and Ti_8_Bi_9_O_0.25_ (this study)

	Ti_8_Bi_9_	Ti_8_Bi_9_O_0.25_
Ti1—Ti1	2.934 (6)	2.992 (2)
Ti1—Ti1	3.074 (3) × 2	3.1142 (19) × 2
Ti1—Ti2	3.017 (2) × 2	3.0228 (6) × 2
Ti1—Bi1	2.971 (4) × 2	2.9610 (9) × 2
Ti1—Bi2	2.848 (1)	2.8305 (11)
Ti1—Bi2	3.074 (5) × 2	3.1175 (6) × 2
Ti1—Bi3	2.945 (4)	2.9491 (11)
Ti2—Bi1	2.848 (1) × 2	2.8488 (2) × 2
Ti2—Bi2	2.931 (1) × 4	2.94278 (11) × 4
Ti3—Bi1	3.122 (6) × 4	3.1227 (16) × 4
Ti3—Bi2	3.144 (6) × 4	3.1434 (16) × 4
Ti4—Bi1	2.937 (5) × 4	2.9398 (13) × 4
Ti4—Bi2	2.985 (5) × 4	2.9771 (13) × 4
O1—Ti1		1.8824 (11) × 4

**Table 2:** Experimental details

Crystal data	
Chemical formula	Ti_8_Bi_9_O_0.25_
*M*_r_	2267.94
Crystal system, space group	Tetragonal, *P*4/*n* *m* *m*
Temperature (K)	301
*a*, *c* (Å)	10.3198 (2), 7.3684 (1)
*V* (Å^3^)	784.72 (3)
*Z*	2
Radiation type	Mo *K*α
μ (mm^−1^)	104.26
Crystal size (mm)	0.10 × 0.08 × 0.06

Data collection	
Diffractometer	Bruker APEXII CCD
Absorption correction	Numerical (*SADABS*; Krause *et al.*, 2015 ▸)
*T*_min_, *T*_max_	0.011, 0.075
No. of measured, independent and observed [*I* > 2σ(*I*)] reflections	14767, 1101, 1079
*R*_int_	0.059
(sin θ/λ)_max_ (Å^−1^)	0.833

Refinement	
*R*[*F* ^2^ > 2σ(*F* ^2^)], *wR*(*F* ^2^), *S*	0.020, 0.044, 1.36
No. of reflections	1101
No. of parameters	34
Δρ_max_, Δρ_min_ (e Å^−3^)	1.55, −1.61

Computer programs: *APEX3* and *SAINT* (Bruker, 2015 ▸), *SHELXT* (Sheldrick, 2015*a* ▸), *SHELXL2014* (Sheldrick, 2015*b* ▸), *VESTA* (Momma & Izumi, 2011 ▸) and *publCIF* (Westrip, 2010 ▸).

## supplementary crystallographic information

### Crystal data

**Table:**

Ti_8_Bi_9_O_0.25_	*D*_x_ = 9.598 Mg m^−^^3^
*M**_r_* = 2267.94	Mo *K*α radiation, λ = 0.71073 Å
Tetragonal, *P*4/*n**m**m*	Cell parameters from 9927 reflections
*a* = 10.3198 (2) Å	θ = 2.8–36.3°
*c* = 7.3684 (1) Å	µ = 104.26 mm^−^^1^
*V* = 784.72 (3) Å^3^	*T* = 301 K
*Z* = 2	Granule, black
*F*(000) = 1850	0.10 × 0.08 × 0.06 mm

### Data collection

**Table:**

Bruker APEXII CCD diffractometer	1101 independent reflections
Radiation source: micro focus sealed tube	1079 reflections with *I* > 2σ(*I*)
Detector resolution: 7.4074 pixels mm^-1^	*R*_int_ = 0.059
ω– and φ–scans	θ_max_ = 36.3°, θ_min_ = 2.8°
Absorption correction: numerical (*SADABS*; Krause *et al.*, 2015)	*h* = −17→17
*T*_min_ = 0.011, *T*_max_ = 0.075	*k* = −16→17
14767 measured reflections	*l* = −11→12

### Refinement

**Table:**

Refinement on *F*^2^	0 restraints
Least-squares matrix: full	*w* = 1/[σ^2^(*F*_o_^2^) + 5.5451*P*] where *P* = (*F*_o_^2^ + 2*F*_c_^2^)/3
*R*[*F*^2^ > 2σ(*F*^2^)] = 0.020	(Δ/σ)_max_ < 0.001
*wR*(*F*^2^) = 0.044	Δρ_max_ = 1.55 e Å^−^^3^
*S* = 1.36	Δρ_min_ = −1.61 e Å^−^^3^
1101 reflections	Extinction correction: SHELXL2014 (Sheldrick, 2015b), Fc^*^=kFc[1+0.001xFc^2^λ^3^/sin(2θ)]^-1/4^
34 parameters	Extinction coefficient: 0.00132 (8)

### Special details

**Table:**

Geometry. All esds (except the esd in the dihedral angle between two l.s. planes) are estimated using the full covariance matrix. The cell esds are taken into account individually in the estimation of esds in distances, angles and torsion angles; correlations between esds in cell parameters are only used when they are defined by crystal symmetry. An approximate (isotropic) treatment of cell esds is used for estimating esds involving l.s. planes.

### Fractional atomic coordinates and isotropic or equivalent isotropic displacement parameters (Å^2^)

**Table:**

	*x*	*y*	*z*	*U*_iso_*/*U*_eq_	Occ. (<1)
Ti1	0.2500	0.60505 (11)	0.15508 (15)	0.00985 (18)	
Ti2	0.0000	0.0000	0.0000	0.0082 (2)	
Ti3	0.2500	0.2500	0.0782 (3)	0.0161 (4)	
Ti4	0.2500	0.2500	0.5769 (3)	0.0094 (3)	
Bi1	0.08500 (2)	0.08500 (2)	0.34804 (3)	0.00996 (6)	
Bi2	0.2500	0.01379 (2)	0.80886 (3)	0.00990 (6)	
Bi3	0.7500	0.2500	0.5000	0.01392 (9)	
O1	0.7500	0.2500	0.0000	0.001 (6)*	0.25 (4)

### Atomic displacement parameters (Å^2^)

**Table:**

	*U*^11^	*U*^22^	*U*^33^	*U*^12^	*U*^13^	*U*^23^
Ti1	0.0095 (4)	0.0098 (4)	0.0102 (4)	0.000	0.000	−0.0005 (3)
Ti2	0.0073 (3)	0.0073 (3)	0.0099 (6)	0.0006 (4)	0.0002 (3)	0.0002 (3)
Ti3	0.0180 (6)	0.0180 (6)	0.0122 (9)	0.000	0.000	0.000
Ti4	0.0081 (5)	0.0081 (5)	0.0119 (8)	0.000	0.000	0.000
Bi1	0.01015 (7)	0.01015 (7)	0.00956 (10)	−0.00054 (6)	−0.00099 (4)	−0.00099 (4)
Bi2	0.00785 (9)	0.01194 (10)	0.00990 (10)	0.000	0.000	0.00110 (6)
Bi3	0.01626 (13)	0.01626 (13)	0.00924 (17)	0.000	0.000	0.000

### Geometric parameters (Å, º)

**Table:**

Ti1—O1^i^	1.8824 (11)	Ti4—Bi1^v^	2.9398 (13)
Ti1—Bi2^ii^	2.8305 (11)	Ti4—Bi1	2.9399 (13)
Ti1—Bi3^iii^	2.9491 (11)	Ti4—Bi2^xviii^	2.9771 (13)
Ti1—Bi1^iv^	2.9610 (9)	Ti4—Bi2^v^	2.9771 (13)
Ti1—Bi1^v^	2.9610 (9)	Ti4—Bi2^iv^	2.9771 (13)
Ti1—Ti1^vi^	2.992 (2)	Ti4—Bi2	2.9771 (13)
Ti1—Ti2^iv^	3.0228 (6)	Bi1—Ti1^xviii^	2.9610 (9)
Ti1—Ti2^v^	3.0227 (6)	Bi1—Ti1^v^	2.9610 (9)
Ti1—Ti1^vii^	3.1142 (19)	Bi1—Bi1^xiv^	3.3423 (4)
Ti1—Ti1^viii^	3.1142 (19)	Bi1—Bi1^iv^	3.4054 (3)
Ti1—Bi2^ix^	3.1175 (6)	Bi1—Bi1^xviii^	3.4054 (3)
Ti1—Bi2^x^	3.1175 (6)	Bi2—Ti1^xx^	2.8305 (11)
Ti2—Bi1	2.8488 (2)	Bi2—Ti2^xxi^	2.9428 (1)
Ti2—Bi1^xi^	2.8488 (2)	Bi2—Ti2^xxii^	2.9428 (1)
Ti2—Bi2^xii^	2.9428 (1)	Bi2—Ti1^xxiii^	3.1175 (6)
Ti2—Bi2^xiii^	2.9428 (1)	Bi2—Ti1^xxiv^	3.1175 (6)
Ti2—Bi2^xiv^	2.9428 (1)	Bi2—Ti3^xxii^	3.1434 (16)
Ti2—Bi2^xv^	2.9428 (1)	Bi2—Bi2^xviii^	3.4474 (3)
Ti2—Ti1^xvi^	3.0228 (6)	Bi2—Bi2^iv^	3.4474 (3)
Ti2—Ti1^v^	3.0228 (6)	Bi2—Bi3^xxv^	3.5482 (2)
Ti2—Ti1^xvii^	3.0228 (6)	Bi3—Ti1^iv^	2.9491 (11)
Ti2—Ti1^xviii^	3.0228 (6)	Bi3—Ti1^iii^	2.9491 (11)
Ti3—Bi1^v^	3.1227 (16)	Bi3—Ti1^xxvi^	2.9491 (11)
Ti3—Bi1^iv^	3.1227 (16)	Bi3—Ti1^xxvii^	2.9491 (11)
Ti3—Bi1^xviii^	3.1227 (16)	Bi3—Bi2^xxviii^	3.5482 (2)
Ti3—Bi1	3.1228 (16)	Bi3—Bi2^xxv^	3.5482 (2)
Ti3—Bi2^xiii^	3.1434 (16)	Bi3—Bi2^xxix^	3.5482 (2)
Ti3—Bi2^xv^	3.1434 (16)	Bi3—Bi2^xviii^	3.5482 (2)
Ti3—Bi2^ii^	3.1434 (16)	O1—Ti1^xxvii^	1.8824 (11)
Ti3—Bi2^xix^	3.1434 (16)	O1—Ti1^xxx^	1.8824 (11)
Ti4—Bi1^iv^	2.9398 (13)	O1—Ti1^iv^	1.8824 (11)
Ti4—Bi1^xviii^	2.9398 (13)	O1—Ti1^i^	1.8824 (11)
			
O1^i^—Ti1—Bi2^ii^	78.30 (4)	Bi2^xv^—Ti3—Bi2^xix^	66.51 (4)
O1^i^—Ti1—Bi3^iii^	96.90 (4)	Bi2^ii^—Ti3—Bi2^xix^	66.51 (4)
Bi2^ii^—Ti1—Bi3^iii^	175.19 (4)	Bi1^iv^—Ti4—Bi1^xviii^	109.99 (7)
O1^i^—Ti1—Bi1^iv^	144.872 (13)	Bi1^iv^—Ti4—Bi1^v^	70.79 (4)
Bi2^ii^—Ti1—Bi1^iv^	98.38 (3)	Bi1^xviii^—Ti4—Bi1^v^	70.79 (4)
Bi3^iii^—Ti1—Bi1^iv^	85.54 (3)	Bi1^iv^—Ti4—Bi1	70.79 (4)
O1^i^—Ti1—Bi1^v^	144.872 (13)	Bi1^xviii^—Ti4—Bi1	70.79 (4)
Bi2^ii^—Ti1—Bi1^v^	98.38 (3)	Bi1^v^—Ti4—Bi1	109.99 (7)
Bi3^iii^—Ti1—Bi1^v^	85.54 (3)	Bi1^iv^—Ti4—Bi2^xviii^	143.472 (1)
Bi1^iv^—Ti1—Bi1^v^	70.21 (3)	Bi1^xviii^—Ti4—Bi2^xviii^	81.667 (5)
O1^i^—Ti1—Ti1^vi^	37.37 (3)	Bi1^v^—Ti4—Bi2^xviii^	81.667 (5)
Bi2^ii^—Ti1—Ti1^vi^	115.67 (2)	Bi1—Ti4—Bi2^xviii^	143.471 (2)
Bi3^iii^—Ti1—Ti1^vi^	59.52 (2)	Bi1^iv^—Ti4—Bi2^v^	81.667 (5)
Bi1^iv^—Ti1—Ti1^vi^	131.482 (19)	Bi1^xviii^—Ti4—Bi2^v^	143.472 (1)
Bi1^v^—Ti1—Ti1^vi^	131.482 (19)	Bi1^v^—Ti4—Bi2^v^	81.667 (5)
O1^i^—Ti1—Ti2^iv^	93.18 (3)	Bi1—Ti4—Bi2^v^	143.471 (1)
Bi2^ii^—Ti1—Ti2^iv^	60.259 (18)	Bi2^xviii^—Ti4—Bi2^v^	70.76 (4)
Bi3^iii^—Ti1—Ti2^iv^	120.510 (18)	Bi1^iv^—Ti4—Bi2^iv^	81.667 (5)
Bi1^iv^—Ti1—Ti2^iv^	56.850 (12)	Bi1^xviii^—Ti4—Bi2^iv^	143.472 (1)
Bi1^v^—Ti1—Ti2^iv^	115.77 (3)	Bi1^v^—Ti4—Bi2^iv^	143.472 (1)
Ti1^vi^—Ti1—Ti2^iv^	111.02 (2)	Bi1—Ti4—Bi2^iv^	81.667 (5)
O1^i^—Ti1—Ti2^v^	93.18 (3)	Bi2^xviii^—Ti4—Bi2^iv^	109.93 (7)
Bi2^ii^—Ti1—Ti2^v^	60.259 (18)	Bi2^v^—Ti4—Bi2^iv^	70.76 (4)
Bi3^iii^—Ti1—Ti2^v^	120.510 (18)	Bi1^iv^—Ti4—Bi2	143.472 (1)
Bi1^iv^—Ti1—Ti2^v^	115.77 (3)	Bi1^xviii^—Ti4—Bi2	81.668 (5)
Bi1^v^—Ti1—Ti2^v^	56.850 (12)	Bi1^v^—Ti4—Bi2	143.472 (2)
Ti1^vi^—Ti1—Ti2^v^	111.02 (2)	Bi1—Ti4—Bi2	81.667 (5)
Ti2^iv^—Ti1—Ti2^v^	117.19 (4)	Bi2^xviii^—Ti4—Bi2	70.76 (4)
O1^i^—Ti1—Ti1^vii^	34.188 (17)	Bi2^v^—Ti4—Bi2	109.93 (7)
Bi2^ii^—Ti1—Ti1^vii^	63.05 (3)	Bi2^iv^—Ti4—Bi2	70.76 (4)
Bi3^iii^—Ti1—Ti1^vii^	112.88 (4)	Ti2—Bi1—Ti4	150.82 (4)
Bi1^iv^—Ti1—Ti1^vii^	113.225 (13)	Ti2—Bi1—Ti1^xviii^	62.668 (16)
Bi1^v^—Ti1—Ti1^vii^	161.22 (5)	Ti4—Bi1—Ti1^xviii^	109.028 (19)
Ti1^vi^—Ti1—Ti1^vii^	61.29 (2)	Ti2—Bi1—Ti1^v^	62.668 (16)
Ti2^iv^—Ti1—Ti1^vii^	58.99 (2)	Ti4—Bi1—Ti1^v^	109.028 (19)
Ti2^v^—Ti1—Ti1^vii^	107.75 (4)	Ti1^xviii^—Bi1—Ti1^v^	122.09 (4)
O1^i^—Ti1—Ti1^viii^	34.188 (17)	Ti2—Bi1—Ti3	76.27 (4)
Bi2^ii^—Ti1—Ti1^viii^	63.05 (3)	Ti4—Bi1—Ti3	74.55 (5)
Bi3^iii^—Ti1—Ti1^viii^	112.88 (4)	Ti1^xviii^—Bi1—Ti3	75.04 (2)
Bi1^iv^—Ti1—Ti1^viii^	161.22 (5)	Ti1^v^—Bi1—Ti3	75.04 (2)
Bi1^v^—Ti1—Ti1^viii^	113.225 (13)	Ti2—Bi1—Bi1^xiv^	106.253 (10)
Ti1^vi^—Ti1—Ti1^viii^	61.29 (2)	Ti4—Bi1—Bi1^xiv^	102.93 (4)
Ti2^iv^—Ti1—Ti1^viii^	107.75 (4)	Ti1^xviii^—Bi1—Bi1^xiv^	106.02 (2)
Ti2^v^—Ti1—Ti1^viii^	58.99 (2)	Ti1^v^—Bi1—Bi1^xiv^	106.02 (2)
Ti1^vii^—Ti1—Ti1^viii^	57.42 (4)	Ti3—Bi1—Bi1^xiv^	177.48 (4)
O1^i^—Ti1—Bi2^ix^	70.76 (2)	Ti2—Bi1—Bi1^iv^	107.934 (3)
Bi2^ii^—Ti1—Bi2^ix^	106.54 (2)	Ti4—Bi1—Bi1^iv^	54.606 (18)
Bi3^iii^—Ti1—Bi2^ix^	71.53 (2)	Ti1^xviii^—Bi1—Bi1^iv^	54.897 (13)
Bi1^iv^—Ti1—Bi2^ix^	141.17 (3)	Ti1^v^—Bi1—Bi1^iv^	131.480 (19)
Bi1^v^—Ti1—Bi2^ix^	76.984 (10)	Ti3—Bi1—Bi1^iv^	56.957 (19)
Ti1^vi^—Ti1—Bi2^ix^	61.325 (18)	Bi1^xiv^—Bi1—Bi1^iv^	121.663 (4)
Ti2^iv^—Ti1—Bi2^ix^	161.73 (4)	Ti2—Bi1—Bi1^xviii^	107.934 (3)
Ti2^v^—Ti1—Bi2^ix^	57.249 (4)	Ti4—Bi1—Bi1^xviii^	54.606 (18)
Ti1^vii^—Ti1—Bi2^ix^	104.57 (2)	Ti1^xviii^—Bi1—Bi1^xviii^	131.480 (19)
Ti1^viii^—Ti1—Bi2^ix^	54.03 (3)	Ti1^v^—Bi1—Bi1^xviii^	54.897 (13)
O1^i^—Ti1—Bi2^x^	70.76 (2)	Ti3—Bi1—Bi1^xviii^	56.957 (19)
Bi2^ii^—Ti1—Bi2^x^	106.54 (2)	Bi1^xiv^—Bi1—Bi1^xviii^	121.663 (4)
Bi3^iii^—Ti1—Bi2^x^	71.53 (2)	Bi1^iv^—Bi1—Bi1^xviii^	90.0
Bi1^iv^—Ti1—Bi2^x^	76.984 (10)	Ti1^xx^—Bi2—Ti2^xxi^	63.110 (6)
Bi1^v^—Ti1—Bi2^x^	141.17 (3)	Ti1^xx^—Bi2—Ti2^xxii^	63.110 (6)
Ti1^vi^—Ti1—Bi2^x^	61.325 (18)	Ti2^xxi^—Bi2—Ti2^xxii^	122.496 (7)
Ti2^iv^—Ti1—Bi2^x^	57.249 (5)	Ti1^xx^—Bi2—Ti4	150.71 (4)
Ti2^v^—Ti1—Bi2^x^	161.73 (4)	Ti2^xxi^—Bi2—Ti4	108.320 (15)
Ti1^vii^—Ti1—Bi2^x^	54.03 (3)	Ti2^xxii^—Bi2—Ti4	108.320 (14)
Ti1^viii^—Ti1—Bi2^x^	104.57 (2)	Ti1^xx^—Bi2—Ti1^xxiii^	62.93 (4)
Bi2^ix^—Ti1—Bi2^x^	121.67 (4)	Ti2^xxi^—Bi2—Ti1^xxiii^	109.73 (2)
Bi1—Ti2—Bi1^xi^	180.0	Ti2^xxii^—Bi2—Ti1^xxiii^	59.756 (18)
Bi1—Ti2—Bi2^xii^	81.606 (5)	Ti4—Bi2—Ti1^xxiii^	139.81 (3)
Bi1^xi^—Ti2—Bi2^xii^	98.393 (5)	Ti1^xx^—Bi2—Ti1^xxiv^	62.93 (4)
Bi1—Ti2—Bi2^xiii^	98.394 (5)	Ti2^xxi^—Bi2—Ti1^xxiv^	59.756 (18)
Bi1^xi^—Ti2—Bi2^xiii^	81.607 (5)	Ti2^xxii^—Bi2—Ti1^xxiv^	109.73 (2)
Bi2^xii^—Ti2—Bi2^xiii^	180.0	Ti4—Bi2—Ti1^xxiv^	139.81 (3)
Bi1—Ti2—Bi2^xiv^	81.606 (5)	Ti1^xxiii^—Bi2—Ti1^xxiv^	57.35 (4)
Bi1^xi^—Ti2—Bi2^xiv^	98.393 (5)	Ti1^xx^—Bi2—Ti3^xxii^	76.52 (4)
Bi2^xii^—Ti2—Bi2^xiv^	71.710 (9)	Ti2^xxi^—Bi2—Ti3^xxii^	74.653 (16)
Bi2^xiii^—Ti2—Bi2^xiv^	108.290 (9)	Ti2^xxii^—Bi2—Ti3^xxii^	74.653 (16)
Bi1—Ti2—Bi2^xv^	98.394 (5)	Ti4—Bi2—Ti3^xxii^	74.19 (5)
Bi1^xi^—Ti2—Bi2^xv^	81.607 (5)	Ti1^xxiii^—Bi2—Ti3^xxii^	128.56 (3)
Bi2^xii^—Ti2—Bi2^xv^	108.290 (9)	Ti1^xxiv^—Bi2—Ti3^xxii^	128.56 (3)
Bi2^xiii^—Ti2—Bi2^xv^	71.710 (9)	Ti1^xx^—Bi2—Bi2^xviii^	107.841 (15)
Bi2^xiv^—Ti2—Bi2^xv^	180.0	Ti2^xxi^—Bi2—Bi2^xviii^	54.145 (4)
Bi1—Ti2—Ti1^xvi^	119.52 (2)	Ti2^xxii^—Bi2—Bi2^xviii^	130.853 (4)
Bi1^xi^—Ti2—Ti1^xvi^	60.48 (2)	Ti4—Bi2—Bi2^xviii^	54.621 (18)
Bi2^xii^—Ti2—Ti1^xvi^	117.005 (19)	Ti1^xxiii^—Bi2—Bi2^xviii^	163.087 (18)
Bi2^xiii^—Ti2—Ti1^xvi^	62.995 (19)	Ti1^xxiv^—Bi2—Bi2^xviii^	106.151 (18)
Bi2^xiv^—Ti2—Ti1^xvi^	56.632 (18)	Ti3^xxii^—Bi2—Bi2^xviii^	56.746 (19)
Bi2^xv^—Ti2—Ti1^xvi^	123.369 (18)	Ti1^xx^—Bi2—Bi2^iv^	107.841 (15)
Bi1—Ti2—Ti1^v^	60.48 (2)	Ti2^xxi^—Bi2—Bi2^iv^	130.853 (4)
Bi1^xi^—Ti2—Ti1^v^	119.52 (2)	Ti2^xxii^—Bi2—Bi2^iv^	54.145 (4)
Bi2^xii^—Ti2—Ti1^v^	62.995 (19)	Ti4—Bi2—Bi2^iv^	54.621 (18)
Bi2^xiii^—Ti2—Ti1^v^	117.005 (19)	Ti1^xxiii^—Bi2—Bi2^iv^	106.151 (18)
Bi2^xiv^—Ti2—Ti1^v^	123.369 (18)	Ti1^xxiv^—Bi2—Bi2^iv^	163.087 (18)
Bi2^xv^—Ti2—Ti1^v^	56.632 (18)	Ti3^xxii^—Bi2—Bi2^iv^	56.746 (19)
Ti1^xvi^—Ti2—Ti1^v^	180.00 (4)	Bi2^xviii^—Bi2—Bi2^iv^	90.0
Bi1—Ti2—Ti1^xvii^	119.52 (2)	Ti1^xx^—Bi2—Bi3^xxv^	104.22 (2)
Bi1^xi^—Ti2—Ti1^xvii^	60.48 (2)	Ti2^xxi^—Bi2—Bi3^xxv^	105.657 (5)
Bi2^xii^—Ti2—Ti1^xvii^	56.631 (18)	Ti2^xxii^—Bi2—Bi3^xxv^	105.657 (5)
Bi2^xiii^—Ti2—Ti1^xvii^	123.369 (18)	Ti4—Bi2—Bi3^xxv^	105.07 (4)
Bi2^xiv^—Ti2—Ti1^xvii^	117.005 (19)	Ti1^xxiii^—Bi2—Bi3^xxv^	52.029 (19)
Bi2^xv^—Ti2—Ti1^xvii^	62.995 (19)	Ti1^xxiv^—Bi2—Bi3^xxv^	52.029 (19)
Ti1^xvi^—Ti2—Ti1^xvii^	117.99 (4)	Ti3^xxii^—Bi2—Bi3^xxv^	179.25 (4)
Ti1^v^—Ti2—Ti1^xvii^	62.01 (4)	Bi2^xviii^—Bi2—Bi3^xxv^	122.853 (2)
Bi1—Ti2—Ti1^xviii^	60.48 (2)	Bi2^iv^—Bi2—Bi3^xxv^	122.853 (2)
Bi1^xi^—Ti2—Ti1^xviii^	119.52 (2)	Ti1^iv^—Bi3—Ti1^iii^	137.96 (3)
Bi2^xii^—Ti2—Ti1^xviii^	123.369 (18)	Ti1^iv^—Bi3—Ti1^xxvi^	137.96 (3)
Bi2^xiii^—Ti2—Ti1^xviii^	56.631 (18)	Ti1^iii^—Bi3—Ti1^xxvi^	60.96 (4)
Bi2^xiv^—Ti2—Ti1^xviii^	62.995 (19)	Ti1^iv^—Bi3—Ti1^xxvii^	60.96 (4)
Bi2^xv^—Ti2—Ti1^xviii^	117.005 (19)	Ti1^iii^—Bi3—Ti1^xxvii^	137.96 (3)
Ti1^xvi^—Ti2—Ti1^xviii^	62.01 (4)	Ti1^xxvi^—Bi3—Ti1^xxvii^	137.96 (3)
Ti1^v^—Ti2—Ti1^xviii^	117.99 (4)	Ti1^iv^—Bi3—Bi2^xxviii^	160.38 (2)
Ti1^xvii^—Ti2—Ti1^xviii^	180.00 (4)	Ti1^iii^—Bi3—Bi2^xxviii^	56.443 (9)
Bi1^v^—Ti3—Bi1^iv^	66.09 (4)	Ti1^xxvi^—Bi3—Bi2^xxviii^	56.443 (9)
Bi1^v^—Ti3—Bi1^xviii^	66.09 (4)	Ti1^xxvii^—Bi3—Bi2^xxviii^	99.42 (2)
Bi1^iv^—Ti3—Bi1^xviii^	100.91 (7)	Ti1^iv^—Bi3—Bi2^xxv^	56.443 (9)
Bi1^v^—Ti3—Bi1	100.91 (7)	Ti1^iii^—Bi3—Bi2^xxv^	160.38 (2)
Bi1^iv^—Ti3—Bi1	66.09 (4)	Ti1^xxvi^—Bi3—Bi2^xxv^	99.42 (2)
Bi1^xviii^—Ti3—Bi1	66.09 (4)	Ti1^xxvii^—Bi3—Bi2^xxv^	56.443 (9)
Bi1^v^—Ti3—Bi2^xiii^	145.571 (2)	Bi2^xxviii^—Bi3—Bi2^xxv^	114.292 (4)
Bi1^iv^—Ti3—Bi2^xiii^	88.805 (5)	Ti1^iv^—Bi3—Bi2^xxix^	56.443 (9)
Bi1^xviii^—Ti3—Bi2^xiii^	145.571 (2)	Ti1^iii^—Bi3—Bi2^xxix^	99.42 (2)
Bi1—Ti3—Bi2^xiii^	88.805 (5)	Ti1^xxvi^—Bi3—Bi2^xxix^	160.38 (2)
Bi1^v^—Ti3—Bi2^xv^	145.571 (2)	Ti1^xxvii^—Bi3—Bi2^xxix^	56.443 (9)
Bi1^iv^—Ti3—Bi2^xv^	145.571 (2)	Bi2^xxviii^—Bi3—Bi2^xxix^	114.292 (4)
Bi1^xviii^—Ti3—Bi2^xv^	88.805 (5)	Bi2^xxv^—Bi3—Bi2^xxix^	100.208 (7)
Bi1—Ti3—Bi2^xv^	88.805 (5)	Ti1^iv^—Bi3—Bi2^xviii^	99.42 (2)
Bi2^xiii^—Ti3—Bi2^xv^	66.51 (4)	Ti1^iii^—Bi3—Bi2^xviii^	56.443 (9)
Bi1^v^—Ti3—Bi2^ii^	88.805 (5)	Ti1^xxvi^—Bi3—Bi2^xviii^	56.443 (9)
Bi1^iv^—Ti3—Bi2^ii^	88.805 (5)	Ti1^xxvii^—Bi3—Bi2^xviii^	160.38 (2)
Bi1^xviii^—Ti3—Bi2^ii^	145.571 (2)	Bi2^xxviii^—Bi3—Bi2^xviii^	100.208 (7)
Bi1—Ti3—Bi2^ii^	145.571 (2)	Bi2^xxv^—Bi3—Bi2^xviii^	114.292 (4)
Bi2^xiii^—Ti3—Bi2^ii^	66.51 (4)	Bi2^xxix^—Bi3—Bi2^xviii^	114.292 (4)
Bi2^xv^—Ti3—Bi2^ii^	101.70 (7)	Ti1^xxvii^—O1—Ti1^xxx^	111.62 (3)
Bi1^v^—Ti3—Bi2^xix^	88.805 (5)	Ti1^xxvii^—O1—Ti1^iv^	105.25 (7)
Bi1^iv^—Ti3—Bi2^xix^	145.571 (2)	Ti1^xxx^—O1—Ti1^iv^	111.62 (3)
Bi1^xviii^—Ti3—Bi2^xix^	88.805 (5)	Ti1^xxvii^—O1—Ti1^i^	111.62 (3)
Bi1—Ti3—Bi2^xix^	145.571 (2)	Ti1^xxx^—O1—Ti1^i^	105.25 (7)
Bi2^xiii^—Ti3—Bi2^xix^	101.70 (7)	Ti1^iv^—O1—Ti1^i^	111.62 (3)

Symmetry codes: (i) −*x*+1, −*y*+1, −*z*; (ii) −*x*+1/2, −*y*+1/2, *z*−1; (iii) −*x*+1, −*y*+1, −*z*+1; (iv) *y*, −*x*+1/2, *z*; (v) −*x*+1/2, −*y*+1/2, *z*; (vi) −*x*+1/2, −*y*+3/2, *z*; (vii) *y*−1/2, −*x*+1, −*z*; (viii) −*y*+1, *x*+1/2, −*z*; (ix) *y*+1/2, −*x*+1, −*z*+1; (x) −*y*, *x*+1/2, −*z*+1; (xi) −*x*, −*y*, −*z*; (xii) −*y*, *x*−1/2, −*z*+1; (xiii) *y*, −*x*+1/2, *z*−1; (xiv) −*x*, −*y*, −*z*+1; (xv) *x*, *y*, *z*−1; (xvi) *x*−1/2, *y*−1/2, −*z*; (xvii) *y*−1/2, −*x*, −*z*; (xviii) −*y*+1/2, *x*, *z*; (xix) −*y*+1/2, *x*, *z*−1; (xx) −*x*+1/2, −*y*+1/2, *z*+1; (xxi) −*y*+1/2, *x*, *z*+1; (xxii) *x*, *y*, *z*+1; (xxiii) *y*−1/2, −*x*, −*z*+1; (xxiv) −*y*+1, *x*−1/2, −*z*+1; (xxv) −*x*+1, −*y*, −*z*+1; (xxvi) *x*+1/2, *y*−1/2, −*z*+1; (xxvii) −*y*+3/2, *x*, *z*; (xxviii) *y*+1, −*x*+1/2, *z*; (xxix) *x*+1/2, *y*+1/2, −*z*+1; (xxx) *x*+1/2, *y*−1/2, −*z*.

## References

[bb1] Amano, S., Bogdanovski, D., Yamane, H., Terauchi, M. & Dronskowski, R. (2016). *Angew. Chem. Int. Ed.* **55**, 1652–1657. 10.1002/anie.201510479 26666574

[bb2] Amano, S. & Yamane, H. (2017). *Inorg. Chem.* **56**, 11610–11618. 10.1021/acs.inorgchem.7b01538 28915024

[bb3] Brese, N. E. & O’Keeffe, M. (1991). *Acta Cryst.* B**47**, 192–197.

[bb4] Bruker (2015). *APEX3* and *SAINT.* Bruker AXS Inc. Madison, Wisconsin, USA.

[bb5] Krause, L., Herbst-Irmer, R., Sheldrick, G. M. & Stalke, D. (2015). *J. Appl. Cryst.* **48**, 3–10. 10.1107/S1600576714022985 PMC4453166 26089746

[bb6] Maruyama, S., Kado, Y. & Uda, T. (2013). *J. Phase Equilib. Diffus.* **34**, 289–296.

[bb7] Momma, K. & Izumi, F. (2011). *J. Appl. Cryst.* **44**, 1272–1276.

[bb8] Okamoto, H. (2010). *J. Phase Equilib. Diffus.* **31**, 314–315.

[bb9] Okamoto, H. (2015). *J. Phase Equilib. Diffus.* **36**, 644–655.

[bb10] Richter, C. G. & Jeitschko, W. (1997). *J. Solid State Chem.* **134**, 26–30.

[bb11] Shannon, R. D. (1976). *Acta Cryst.* A**32**, 751–767.

[bb12] Sheldrick, G. M. (2015*a*). *Acta Cryst.* A**71**, 3–8.

[bb13] Sheldrick, G. M. (2015*b*). *Acta Cryst.* C**71**, 3–8.

[bb14] Westrip, S. P. (2010). *J. Appl. Cryst.* **43**, 920–925.

[bb15] Yamane, H. & Amano, S. (2017). *J. Alloys Compd.* **701**, 967–974.

